# Mesenchymal stromal cell derived CCL2 is required for accelerated wound healing

**DOI:** 10.1038/s41598-020-59174-1

**Published:** 2020-02-14

**Authors:** Derek S. Whelan, Noel M. Caplice, Anthony J. P. Clover

**Affiliations:** 1 Centre for Research in Vascular Biology, University College Cork, Cork, Ireland; 2 Department of Plastic Surgery, University College Cork, Cork, Ireland

**Keywords:** Stem-cell biotechnology, Tissue engineering, Mesenchymal stem cells, Translational research

## Abstract

Mesenchymal stromal cells (MSC) have immunomodulatory effects impacting macrophages, promoting polarisation towards a reparative phenotype. CCL2 is a potent cytokine involved in the recruitment of macrophages. We hypothesised that MSC derived CCL2 may be involved in the MSC therapeutic effect by facilitating macrophage repolarisation. To further delineate this mechanism, MSC isolated from CCL2 deficient mice (MSC-KO) were applied to excisional wounds in wild-type (WT) mice. CCL2 deficiency in MSC completely abrogated the therapeutic response compared to MSC-WT. MSC-KO were unable to repolarise macrophages to the same extent as WT and this was accompanied by a reduced angiogenesis and re-epithelialisation of the wounds at day 10. This study demonstrates that MSC derived CCL2 is required for MSC induced accelerated wound healing. The role of CCL2 in the interaction between MSC and Macrophages has not been previously demonstrated in accelerated wound healing. CCL2 has a potent effect on the ability to reduce the inflammatory response through local recruitment of macrophages. This research highlights CCL2 as a possible target for augmentation of MSC therapy to enhance therapeutic potential.

## Introduction

In the regeneration and repair of cutaneous wounds, Mesenchymal Stromal Cell (MSC) therapy holds much promise and several clinical studies have been completed, demonstrating improved wound healing after application of MSC^1^. This therapeutic MSC effect is mediated in part through soluble factors and cell contact mechanisms leading to increased angiogenesis, granulation tissue formation and re-epithelialisation^2,3^.

Several studies have corroborated the therapeutic benefit of MSC therapy in cutaneous wound healing^2–4^, the effects of which, have been observed in mouse^2^ rabbit^5^, rat^6^, pig^7^ and human subjects^1^. The molecular mechanisms behind this accelerated wound healing are still incompletely understood but it is known that MSC therapy accelerates wound closure, involving earlier granulation tissue formation^6^ and increased re-epithelialisation^8^. This healing process is driven by a potent angiogeneic effect which has been observed both *in vivo* and *in vitro*, and is mediated primarily through VEGF secretion^8,9^. MSC are also potent immune-modulators of both adaptive and innate immune systems, and several well-known immune modulating factors have been implicated (TSG-6^10^, IDO or inducible NOS^11^ and PGE2^12^) in altering the inflammatory environment.

MSC are involved in a phenotypic switch of inflammatory macrophages (M1) towards a reparative type phenotype (M2) associated with reduced TNFα and increased IL-10 production^13^ thereby accelerating wound healing progression^4^. It is believed this process is mediated through soluble immunomodulatory factors and cell–cell contact.

The exact mechanisms involved remain poorly understood, and it is not known if multiple cytokines are involved in facilitating macrophage recruitment or if there is cell-cell crosstalk between the macrophages and MSC that facilitates the process. Improved understanding of cytokine regulation and optimal MSC function may augment MSC healing and therapeutic potential.

CCL2 is a chemokine classically associated with the recruitment of macrophages and monocytes during angiogenesis^14,15^. CCL2 is also required for normal progression of wound healing phases, CCL2 KO mice had reduced re-epithelialisation associated with reduced macrophage infiltration of wounds^14^.

Under unstimulated conditions, MSC secrete CCL2 (0.2–2 ng/ml)^4,16^. When stimulated with inflammatory cytokines such as TNFα (which is present within the wound), MSC secrete up to 10 fold more CCL2 and were found to recruit Ly6C hi CD11b monocytes, F4/80 macrophages and CD11b + Ly6G + neutrophils in a CCR-2 dependent manner *in vivo*^17^. When cultured with macrophage condition media (CM), MSC displayed an altered gene expression with a fourfold increase in CCL2 expression. MSC CM enhanced monocyte migration and this could be attenuated with magnetic bead CCL2 depletion of MSC CM^18^.

The role of CCL2 has not been previously clearly established in MSC accelerated wound healing. Despite a wealth of literature demonstrating CCL2 as a potent inducer of the recruitment of monocytes/macrophages, its role in MSC modulation of wound healing is still not clear due in part to the complexity and redundancy of downstream signalling cascades, posttranslational modifications and decoy receptors involved in CC signalling^19^. We choose to study the role of CCL2 as it has both angiogenic and immune modulatory functions, two of the key therapeutic mechanisms that have been identified in MSC accelerated wound healing. CCL2 has several angiogenic properties, It can induce endothelial cell proliferation and migration through CCR2^20,21^. In addition, treatment of human endothelial cells with CCL2 induces hypoxia inducible factor 1 gene expression this in turn induces VEGF^22^. CCL2 can also induce expression of tissue factor, a potent clotting factor, on the cell surface of vascular smooth muscle cells^23^. This in combination with its well established role in macrophage recruitment made it a very interesting candidate whose role in MSC accelerated wound healing had not been previously studied.

We hypothesised that CCL2 participates in the local recruitment of macrophages and facilitates their re-polarisation. To test this hypothesis, MSC were isolated from either wild type mice (MSC-WT) or CCL2 KO mice (MSC-KO) and used in splinted excisional wound healing experiments to compare therapeutic efficacy.

## Clinical Problem Addressed

Slow wound healing has an impact on health care system. The resulting scars from wounds have both physical and mental impacts that can greatly affect quality of life. Reductions in severity and duration of inflammatory responses can result in reduced scar appearance and formation and may attenuate hypertrophic scarring.

MSC have shown promise in various animal studies however further optimisation is required. One of the primary mechanisms in MSC therapy is their ability to reduce inflammatory response. This is believed to be through cell contact mechanism. Greater understanding of the key molecules involved in this process allows further refinement of the treatment, where MSC can be engineered to over express these various factors leading to increased resolution of inflammatory response and accelerated wound closure.

## Materials and Methods

### MSC isolation and cell culture

Primary MSC were isolated from female C57 mice (8–12 wk old) as per protocol published by Soleimani *et al*.^24^. The cell suspension was filtered and plated at a density of 25 × 10^6^ cells/ml in α-MEM media (Sigma, Wicklow, Ireland) supplemented with 10% FBS (Thermo Fisher, Waltham, Ma) and 1x antibiotic/antimycotic. Cells were then passaged and further expanded up to P5 prior to characterisation of differentiation capacity and expression of cell surface antigens and subsequent experimental use.

#### Isolation of murine CCL2 KO MSC

Using the same protocol BM-MSC were isolated from 8–12 wk old female transgenic CCL2 knock-out mice obtained from JAX laboratories (JAX laboratories, Bar Harbour, ME) and referred to as MSC-KO. CCL2 deficiency was validated by enzyme-linked immunosorbent assay (ELISA) (RD Systems Minneapolis, MN).

### Macrophage MSC co-culture

MSC (P5) were seeded in a 24 well plates at a concentration of 1 × 10^5^/well. The next day, 1 × 10^5^ macrophages (RAW 264.7 cells obtained from ATTC, Manassas, VA) per well were seeded with MSC. After 24 h culture, macrophages were stimulated with LPS (Sigma, Wicklow, Ireland) (30 ng/ml). Culture supernatants were collected after 48 h and centrifuged at 2000 g to remove any cell debris before storing at −20 °C prior to analysis of TNFα concentration by ELISA (RD Systems Minneapolis, MN).

### Transwell migration assay

MSC were seeded in the bottom of a transwell chamber at a density of 2.5 × 10^4^ and incubated overnight. RAW 264.7 cells were then seeded in the top chamber of an 8 μm transwell insert (Corning, New York, NY). Growth media was replaced with serum free media and incubated for 12 h. Transwell inserts were then removed and rinsed in PBS (Sigma, Wicklow, Ireland). A cotton bud was used to remove un-migrated cells from upper side of membrane. Membranes were then stained with Hoechst (Thermo Fisher Scientific, Rochford, UK) stain for 15 min and imaged (Nikon eclipse TE2000-E, Tokyo, Japan) at five random locations and migrated cells quantified (Nis Elements v3.0, Nikon, Tokyo, Japan).

### Mouse wound healing model

All animal experiments performed were performed in accordance with relevant guidelines and regulations and were approved by University College Cork Animal Experimentation Ethics Committee and by the Health Product Regulatory Authority, the regulatory body for experimental animal studies in Ireland.

This protocol was previously described in detail by Wang *et al*.^25^. Wild type mice were anaesthetised by an IP injection of Ketamine (90 mg/kg) (Vetalar, Boehringer, Ingelheim Germany) and Xylazine (10 mg/kg) (Sedaxylan, Bimeda, Dublin, Ireland) and skin was sterilised with 70% (vol/vol) ethanol swab. The skin on the dorsum was extended and two 5 mm punch wounds were induced. To evaluate wound closure, images of the wounds were taken at days 0, 3, 5, 7 and 10 using a DLSR camera (Canon, Tokyo, Japan). A metric ruler was included in every image to allow for normalisation and distance calibration. Wound area was then calculated using Image J software (NIH image software). Mice were randomised into either Control, MSC-WT or MSC-KO treated groups. Wounds were injected intradermally using a 27 g needle (Beckton and Dickinson Franklin Lakes NJ) at four points around the edge of the wound with either a total of 1 × 10^6^ MSC in 100 μl PBS or just 100 μl PBS for controls. Intradermal injection is widely used to delivery therapeutics in the mouse model and it did not cause significant injury to the epidermis surrounding the wound. A silicone splint was used to prevent fibrotic contraction of the wound. The silicone ring was attached with an adhesive and then sutured into place. The wounds were covered with a non-occlusive dressing (Tegaderm, 3M, Maplewood, MN) and then covered with a self-adhering elastic bandage (Optitape, Smith and Nephew, London, UK).

Dressings were changed and wounds imaged on days 3, 5, 7, and 10. On day 10, the mice were euthanised by CO_2_ administration and wounds were harvested for further analysis.

### Statistical analysis

Difference between multiple groups was analysed by analysis of variance (ANOVA) followed by Tukey’s post-hoc testing. Results are expressed as mean ± SEM. All statistics were carried out using Graphpad Prism v7.03.

## Results

### Isolation and characterisation of MSC from WT and CCL2 knock-out mice

MSC were fully characterised as plastic adherent with tri differentiation capacity and expressed defined cell surface antigen profile (Supplementary Figs. 1, 2). ELISA was performed on culture supernatants to validate that MSC-WT secreted CCL2 under normal conditions and MSC-KO did not express CCL2 (Supplementary Fig. 3a).

### CCL2 deficient MSC have reduced therapeutic efficacy in excisional mouse model

**Figure 1:**
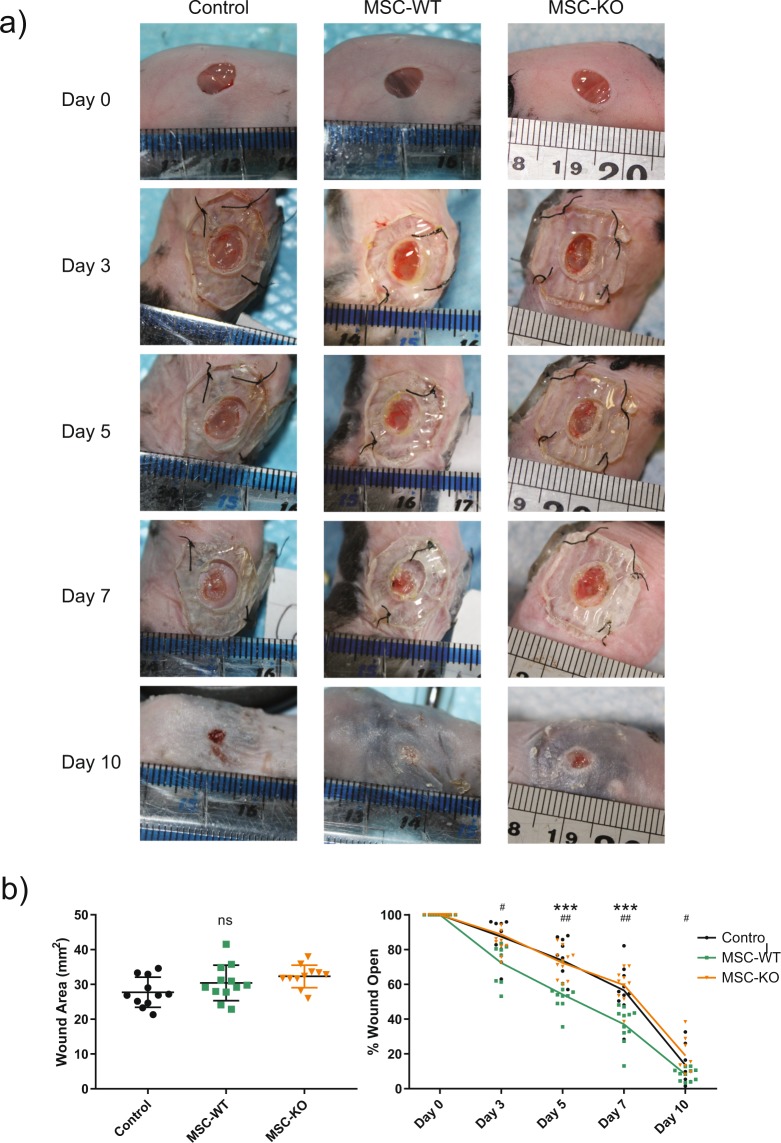
CCL2 deficiency reduces therapeutic efficacy of MSC in cutaneous wound healing. (**a)** Images of mouse excisional wounds between d-0 and d-10. Two 5 mm punch wounds were created on the dorsum of each mouse. **(b)** Left - Wound area was quantified at d-0 and no significant differences were observed between any of the groups (n ≥ 11, ns, not significant). (**b)** Right - Time course of wound closure from image analysis demonstrated accelerated wound closure in MSC-WT treated wounds (n ≥ 8, * = MSC-WT vs Control, ***p < 0.001). MSC-KO completely abrogated this therapeutic effect and were not statistically different to control treated wounds, but were statistically different to MSC-WT treated wounds at all time points (n ≥ 8, # = MSC-WT vs MSC-KO, #p < 0.05, ##p < 0.001).

Excisional wounding of mice was carried out and image analysis was performed to calculate the wound size at d-0. No significant differences in wound area were observed at d-0 (Fig. 1b).

Intradermal injection of MSC-WT accelerated wound closure at all time points as indicated by temporal analysis over 10 days and statistically significant differences were observed from day 3 onwards compared to Controls (Fig. 1b). Strikingly MSC-KO did not accelerate wound closure and were not statistically different to controls at any time point (**Day 3** MSC-WT vs Control, p = 0.053; MSC-WT vs MSC-KO p = 0.034. **Day 5** Control vs MSC-WT p < 0.001, Control vs MSC-KO p = 0.945, MSC-WT vs MSC-KO p < 0.001). **Day 7** Control vs MSC p < 0.001, Control vs MSC-KO p = 0.828, MSC-WT vs MSC-KO p < 0.001. **Day 10** Control vs MSC-WT p = 0.390, Control vs MSC-KO p = 0.425, MSC-WT vs MSC-KO p = 0.032, n ≥ 8).

### MSC-KO do not enhance re-epithelialisation of excisional wounds

**Figure 2:**
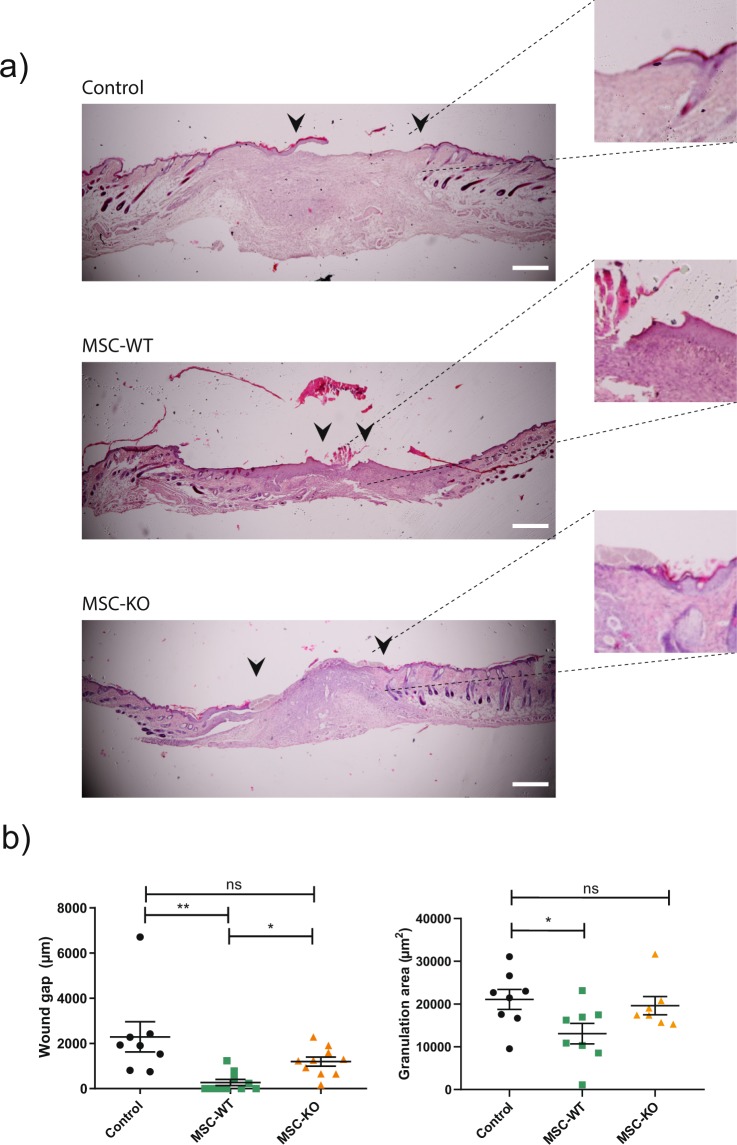
CCL2 deficiency delays wound re-epithelialisation compared to MSC-WT. (**a)** Histological samples taken at d-10 post wounding were stained with H&E. Images of stained tissue sections were taken at 4×. Black arrows indicate migrating front of Keratinocytes; insets show magnified image of migrating front. Scale bar, 100 μm. (**b)** Left- Quantification of unepithelialised gap demonstrated significantly reduced unepithelialised wound in MSC-WT treated wounds compared to both control and MSC-KO (n ≥ 8 ns, not significant *p < 0.05, **p < 0.01). (**b)** Right - Granulation area was measured using ImageJ software indicated significantly reduced area in MSC-WT treated wounds compared to control suggestive of reduced inflammatory response and increased wound maturity (n ≥ 7 ns, not significant, *p < 0.05).

Application of MSC-WT accelerated re-epithelialisation (Fig. 2a) as measured on d-10 (Control vs MSC-WT p = 0.002, Control vs MSC-KO p = 0.11, MSC-WT vs MSC-KO p = 0.165, n ≥ 8). Several MSC-WT treated wounds were fully re-epithelialised at the time of analysis. Epithelial gap was reduced in MSC-KO group but, this was not statistically different to Control.

Granulation area was also measured (Fig. 2b) and found to be largest in the control group, Control vs MSC-WT p = 0.011, Control vs MSC-KO p = 0.606, MSC-WT vs MSC-KO p = 0.097, n ≥ 8). MSC-WT treated wounds had statistically smaller granulation area indicating resolution of inflammatory response and increased wound maturation.

### MSC derived CCL2 is required for reduced inflammatory response and accumulation of CD206+ macrophages

**Figure 3:**
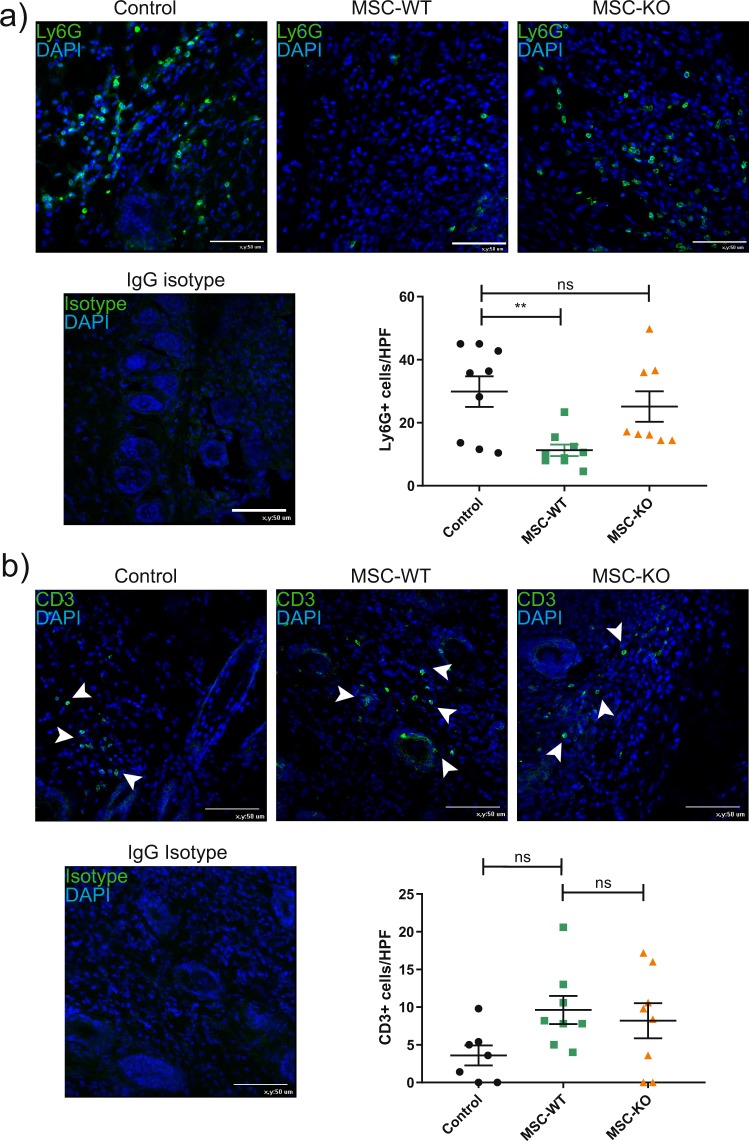
MSC alter innate immune response in CCL2 dependent mechanism. (**a**) Representative images of wound sections d-10 post wounding, stained for neutrophil marker Ly6G (green), cell nuclei were counter stained with DAPI (blue) scale bar, 50 μm. MSC-WT treated wounds had significantly reduced neutrophil infiltration at d-10 compared to control and MSC–KO treated wounds (n ≥ 8, ns, not significant **p < 0.01). (**b**) Representative images of wound sections d-10 post wounding, stained for T-cell marker CD3 (green), cell nuclei stained with DAPI (blue) scale bar, 50 μm. Both MSC-WT and MSC-KO treated wounds had a trend towards increased CD3+ T-cells within the wound bed at d-10 however this was not statistically different to controls (n ≥ 7, ns, not significant).

To characterise neutrophil response, d-10 wound sections were examined by immunohistochemistry for the neutrophil marker Ly6G (Fig. 3a). Wounds treated with MSC-WT showed a significant reduction in Ly6G+ neutrophil infiltration compared to PBS treated controls (Control vs MSC-WT, p < 0.001, Control vs MSC-KO, p = 0.435, MSC-WT vs MSC-KO, p = 0.009, n ≥ 8). While MSC-KO have a significant amount Ly6G+ positive cells remaining within the wound similar to control wounds. Sustained neutrophil presence can indicate ongoing inflammatory response, possibly due to lack of a complete epithelium^26^.

The pan T-Cell marker CD3, was used to evaluate T-cell presence within the wound (Fig. 3b). As expected T-cell numbers are relatively low at d-10, however there is a trend towards greater CD3+ T-cells in both MSC-WT and MSC-KO groups compared to the control group (Control vs MSC-WT, p = 0.1, Control vs MSC-KO, p = 0.246, MSC-WT vs MSC-KO, p = 0.857, n ≥ 7). While this was not statistically significant, it may indicate that T-cell infiltration is driven by MSC but is not CCL2 dependent.

**Figure 4:**
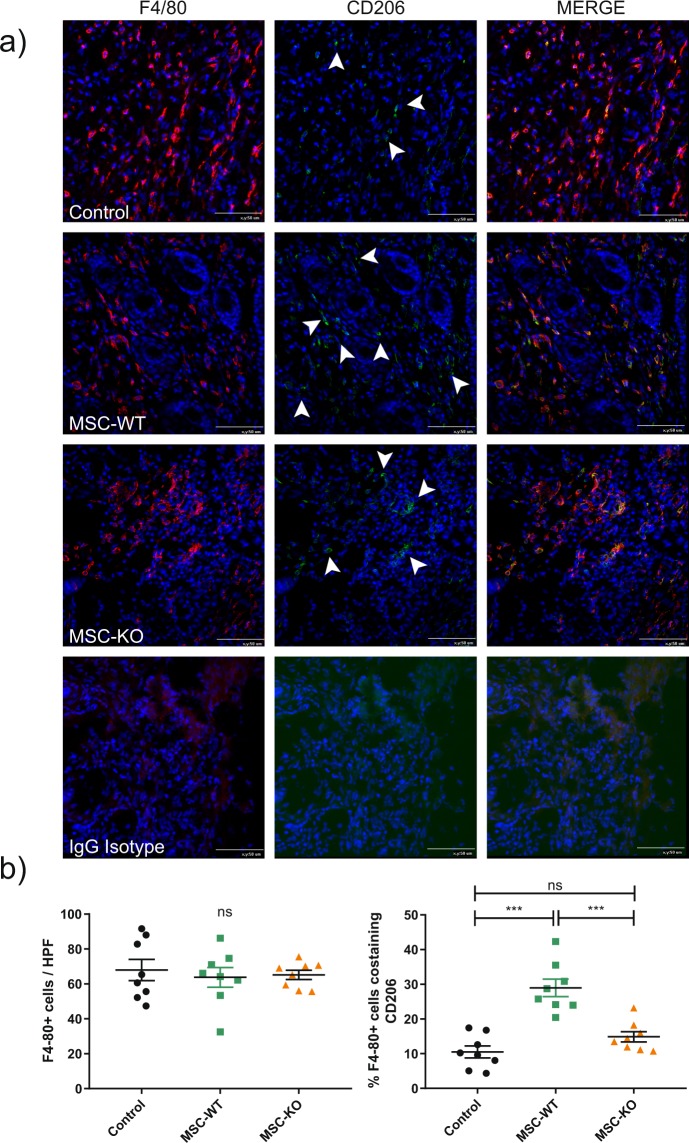
MSC derived CCL2 is required for increased M2 polarisation of macrophages within the wound. (**a**) Representative images of wound sections d-10, stained for pan macrophage marker F4/80 (red) and M2 marker CD206 (green), cell nuclei were counter stained with DAPI (blue), for clarity arrowheads indicate CD206+ cells, scale bar 50 μm. **(b)** Left- total macrophage within the wound bed, no difference between any of the groups was detected at d-10 (n ≥ 8 ns, not significant). **(b)** Right- percentage macrophages co-expressing CD206 was significantly increased in MSC-WT treated wounds compared to both control and MSC-KO treated wounds. (n ≥ 8 ns, not significant ***p < 0.001).

To investigate the response towards MSC derived CCL2, immunohistochemistry was performed on tissues sections for pan macrophage marker F4/80 (red). To determine the polarisation of macrophages towards reparative M2 phenotype, sections were co-stained with CD206 (green). Quantification was performed of positive cells within the wound bed (Fig. 4). No significant difference in macrophages numbers was observed within the wound between treatment groups (Control vs MSC-WT p = 0.826, Control vs MSC-KO p = 0.92, MSC-WT vs MSC-KO p = 0.98, n ≥ 8). However, the frequency of CD206+ macrophages was greatly increased in MSC-WT treated group (Control vs MSC p < 0.001, Control vs MSC-KO p = 0.277, MSC-WT vs MSC-KO p < 0.001, n ≥ 8). MSC-KO treated wounds had significantly less CD206+ macrophages compared to MSC-WT group. There was no significant difference between MSC-KO and control groups.

To further characterise the inflammatory cytokine profile of the wound, qRT-PCR was performed on wound samples from d-10 (Supplementary Fig. 4). Significantly, there was a strong increase with IL-10 expression with MSC-WT treated wounds compared to both control and MSC-KO treated wounds (Control vs MSC-WT p = 0.037, Control vs MSC-KO p = 0.998, MSC-WT vs MSC-KO p = 0.02, n = 5). There was no significant difference between MSC-KO and control, groups.

### CCL2 deficient MSC have reduced immunomodulatory capacity

**Figure 5:**
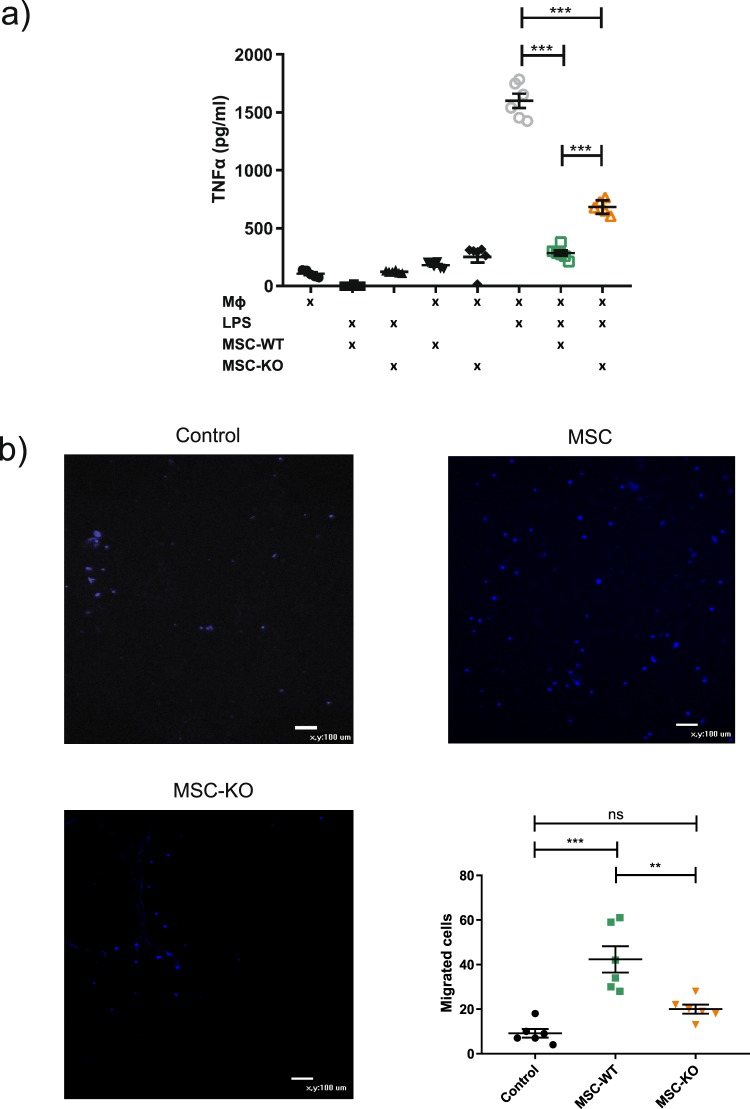
CCL2 deficiency reduced MSC immunomodulatory capacity. (**a**) Co-culture of MSC-WT in direct cell contact with stimulated macrophages (LPS 30 ng/ml) significantly reduced TNFα as detected by ELISA in culture supernatants at 48 h. MSC-KO were also able to reduce TNFα however, not to the same extent as MSC-WT (n = 6 ***p < 0.001). (**b**) Transwell migration assay, Images of migrated macrophages (stained with Hoechst live stain, blue) demonstrate significantly reduced migration towards MSC-KO compared to WT (n = 6 **p < 0.01, ***p < 0.001).

To verify *in vivo* results that CCL2 is required for efficient M2 polarisation, MSC-WT and MSC-KO were co-cultured with LPS stimulated (30 ng/ml) macrophages and supernatant concentrations of TNFα and IL-10 were assessed (Fig. 5a).

In response to LPS stimulation of macrophages significantly increased TNFα secretion associated with activated M1 macrophage phenotype (Mϕ + LPS vs Mϕ p < 0.001 n = 6). Co-culture of MSC-WT significantly reduced TNFα to baseline secretion consistent with increased M2 polarisation and this was only observed when cultured with stimulated macrophages (MSC-WT + Mϕ + LPS vs Mϕ + LPS p < 0.001, n = 6).

MSC-KO were not as efficient as MSC-WT in reducing TNFα and associated M2 polarisation as significantly greater concentrations of TNFα were detected in this group (MSC-KO + Mϕ + LPS vs MSC-WT + Mϕ + LPS, p < 0.001, n = 6). MSC-KO were, however, able to induce a statistically significant reduction in TNFα, albeit smaller (Mϕ + LPS vs MSC-KO + Mϕ + LPS, p < 0.001, n = 6).

Detection of IL-10 in supernatants (Supplementary Fig. 4) demonstrated both MSC-WT and MSC-KO were capable of increasing IL-10 in response to stimulated macrophages (Mϕ + LPS vs MSC-WT + Mϕ + LPS p < 0.001, Mϕ + LPS vs MSC-KO + Mϕ + LPS p < 0.001, n = 6).

Transwell experiments were used to determine migratory response of macrophages towards MSC in response to CCL2 (Fig. 5b). MSC-WT group had significantly more migrated macrophages compared to control and to MSC-KO group (Control vs MSC-WT, p < 0.001, Control vs MSC-KO p = 0.112, MSC-WT vs MSC-KO, p = 0.01, n = 6).

To determine if MSC alter CCL2 secretion in response to the inflammatory wound environment, MSC were stimulated with various concentrations of TNFα and CCL2 concentration in supernatants was assayed by ELISA. CCL2 was significantly increased upon stimulation of TNFα in a clear dose dependent manner (Supplementary Fig. 3b)

### MSC derived CCL2 is also required for angiogeneic effect

**Figure 6:**
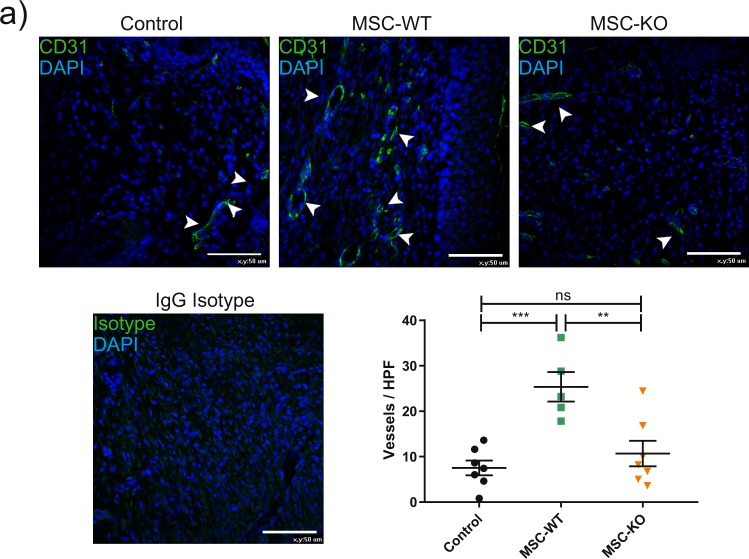
MSC derived CCL2 is also required for angiogeneic response. Representative images of wound sections at d-10 post wounding, probed with CD31 antibody (green) and nuclei counter stained with DAPI (blue) scale bar, 50 μm. Vessels per high power field were quantified within the wound bed, white arrows indicate intact vessel lumens. MSC treated wounds had increased vessel density compared to both control and MSC-KO treated wounds (n ≥ 5 ns, not significant ***p < 0.001).

To determine angiogeneic response, wound sections were stained for CD31 (green) and blood vessel density within the wound bed was calculated (Fig. 6).

Vessel density was significantly improved with application of MSC-WT to the wounds compared to Control (Control vs MSC-WT p < 0.001, Control vs MSC-KO p = 0.632, MSC-WT vs MSC-KO p = 0.003, n ≥ 5). In contrast, CCL2 deficient MSC did not significantly increase vessel density compared to control.

## Discussion

This work demonstrates a novel non-redundant role of MSC derived CCL2 in the M1-M2 polarising mechanism a key feature of MSC therapeutic response. CCL2 deficient MSC were unable to expand the M2 macrophage population within the wound as observed with MSC-WT and there was no significant increase in IL-10 compared to controls. Significantly, our *in vitro* studies confirmed that CCL2 deficient MSC had a reduced capacity to modulate TNFα secretion by stimulated macrophages. To the best of our knowledge, this finding has not been established before.

MSC can modulate both adaptive and innate immune responses based on the micro environmental cues. MSC inhibit the proliferation and activity of B-cells^27^, T-cells^28^ and NK cells though a range of cytokines such as TSG-6 and COX-2 activity leading to downstream PGE2 release. One of the major attributes of MSC therapy in cutaneous wound healing is the ability to polarise macrophage phenotype towards reparative M2 type^4,29^. This is characterised with increased CD206+ macrophages within the wound, reduced inflammatory cytokine TNFα and increased IL-10, as was established *in vitro* in this study. This results in an accelerated progression of wound healing. As the inflammatory phase resolves, M2 macrophages increase fibroblast proliferation, allowing formation of granulation tissue and vascularisation to occur. The addition of MSC expressing CCL2 may not function primarily in recruiting monocytes to the wound, as CCL2 is endogenously expressed within the wound rapidly after injury^30^; it may, however, facilitate local homing and cell contact between recruited monocytes/macrophages and MSC within the wound. Nemeth *et al*. elegantly elucidated the mechanisms of M2 polarisation, in a series of co-culture experiments they demonstrate that a significant increase in IL-10 only occurs when stimulated macrophages are co-cultured with MSC^29^. If cultured in a transwell system, no significant increase in IL-10 is observed, demonstrating the requirement of cell contact to initiate polarisation^29^. Conversely, TNFα was only significantly reduced with direct co-culture of MSC and stimulated macrophages^29^. Other studies have reported similar results^31,32^. In this study, these findings were exactly replicated with MSC-WT. However, MSC-KO were not as efficient in repolarising stimulated macrophages and reducing TNFα, establishing a possible causative mechanism for the reduced efficacy of MSC-KO observed *in vivo*.

In this study, *in vitro* co-culture of MSC-WT was able to significantly increase IL-10 in stimulated macrophages. In contrast to previous findings with TNFα, MSC-KO were also capable of inducing IL-10, which may be IL-6 dependent^33^. The time point of 48 h used in these experiments may also not capture possible early induction of IL-10 by MSC-WT. While no difference in IL-10 was observed *in vitro*, it should be noted that IL-10 was significantly increased in MSC-WT treated wounds and not MSC-KO treated wounds (Supplementary Fig. 5). *In vitro* co-cultures may also not entirely reflect the complex multi cellular environment present *in vivo*.

The *in vivo* data presented here in conjunction with reduced *in vitro* macrophage recruitment and reduced capacity to inhibit TNFα, suggests that CCL2 is required for local contact of MSC and macrophages, which facilitates increased M2 polarisation.

MSC derived CCL2 may also directly influence macrophage polarisation^34^. M1 polarised macrophages isolated from CCR-2 −/− deficient mice show reduced IL-10 expression compared to wild type, while normal M1 when stimulated with CCL2 increased IL-10 secretion^34^.

MSC may also have secondary inhibitory effects based on IL-10 as increased IL-10 from other immune cells, such as macrophages, can inhibit rolling, adhesion and trans-epithelial migration of neutrophils^35^ and may prevent their re-entry into the wound cutting off the negative feedback loop in response to macrophage stimulation. This is consistent with observations in this study, where MSC treated wounds had increased expression of IL-10 and significantly less Ly6G+ neutrophils present compared to MSC-KO and control treated wounds at d-10.

While the M1/M2 model is a useful framework for conceptualising the macrophage driven inflammatory response and resolution thereof. It does have its limitations and can suffer from been overly simplistic and ill defined^36^. Ongoing developments in macrophage biology have further refined the M2 category into several subsets M2a, M2b, and M2c based on the activation molecule and deferential expression of surface markers and secreted factors^37^. These M2 subtypes have been identified and used *in vitro* to study M2 like macrophages with different characteristics. However, while these subsets have been well-defined *in vitro* it is not known if they exist *in vivo*^37,38^.

It is now widely believed that M1 and M2 are not discrete differentiation states but that macrophages have an innate plasticity based on the surrounding environmental cues and exist on a continuum of these states^36,38^. The role of these various subset phenotypes in the stages of wound healing is still poorly defined and more research is required further to identify what subsets provide what functions to provide more granularity to these processes. In this context new models are currently been developed that focus on function such as pro inflammatory- pro wound healing and pro resolving^39,40^.

In mice, wound closure is driven by wound contraction however, in this study a sutured ring prevented this from occurring. Lack of contraction requires granulation tissue to be formed to allow re-epithelisation and wound closure to progress. MSC therapy accelerates the formation of granulation tissue^6,41^ and CCL2 may be involved in this respect. Granulation tissue is a vascular dense connective tissue consisting of a provisional extra cellular matrix of fibronectin, fibrin and other components and is required substrate for keratinocytes to migrate on. Increased angiogenesis in MSC treated wounds accelerate the process of granulation tissue formation, thereby, providing a substrate for keratinocytes to migrate across, leading to augmented re-epithelialisation that was observed in this study.

A significantly increased angiogeneic response in MSC treated wounds manifested in increased vascular density in the wound bed. This phenomenon was reported by several other studies and is now believed to be a major contributor to accelerated wound closure^6,8,42,43^. CCL2 plays a pivotal role in angiogenesis and importantly, no significant increase in vascularisation compared to controls was observed in MSC-KO treated wounds. The role of angiogenesis during wound repair is well established and involves the migration and proliferation of vascular cells. CCL2 induces downstream effects on SMCs and endothelial cells^44^. In addition, endothelial cells express CCR-2 and migrate in response to CCL2^45^. MSC conditioned media can induce an angiogeneic response in endothelial cells and may have a protective effect under hypoxic conditions, which is CCL2 dependent^42^.

In conclusion, this study establishes a novel non-redundant role for MSC derived CCL2 in accelerated wound closure which was found to affect both the vascular, epithelial and inflammatory response within cutaneous wounds. This research highlights CCL2 as a possible target for augmentation of MSC therapy to enhance therapeutic potential.

## Supplementary information

Supplementary information.

## Data Availability

The data generated and analysed during this study are included in this published article and its Supplementary Information Files.
