# Supplementary material for: Mesenchymal stromal cell derived CCL2 is required for accelerated wound healing
Source: Sci Rep. 2020 Feb 14;10:2642. doi: 10.1038/s41598-020-59174-1 (PMC7021763; doi:10.1038/s41598-020-59174-1)
Supplement: Supplementary file 1 — Supplementary information. [file 41598_2020_59174_MOESM1_ESM.pdf]

Supplementary Information:

Mesenchymal stromal cell derived CCL2 is required for accelerated wound healing

Derek S. Whelan, PhD[1], Noel M. Caplice, MD, PhD[1] Anthony JP Clover, MD[1,2]

[1]Centre for Research in Vascular Biology, University College Cork, Cork, Ireland

[2]Department of Plastic Surgery, University College Cork, Cork, Ireland

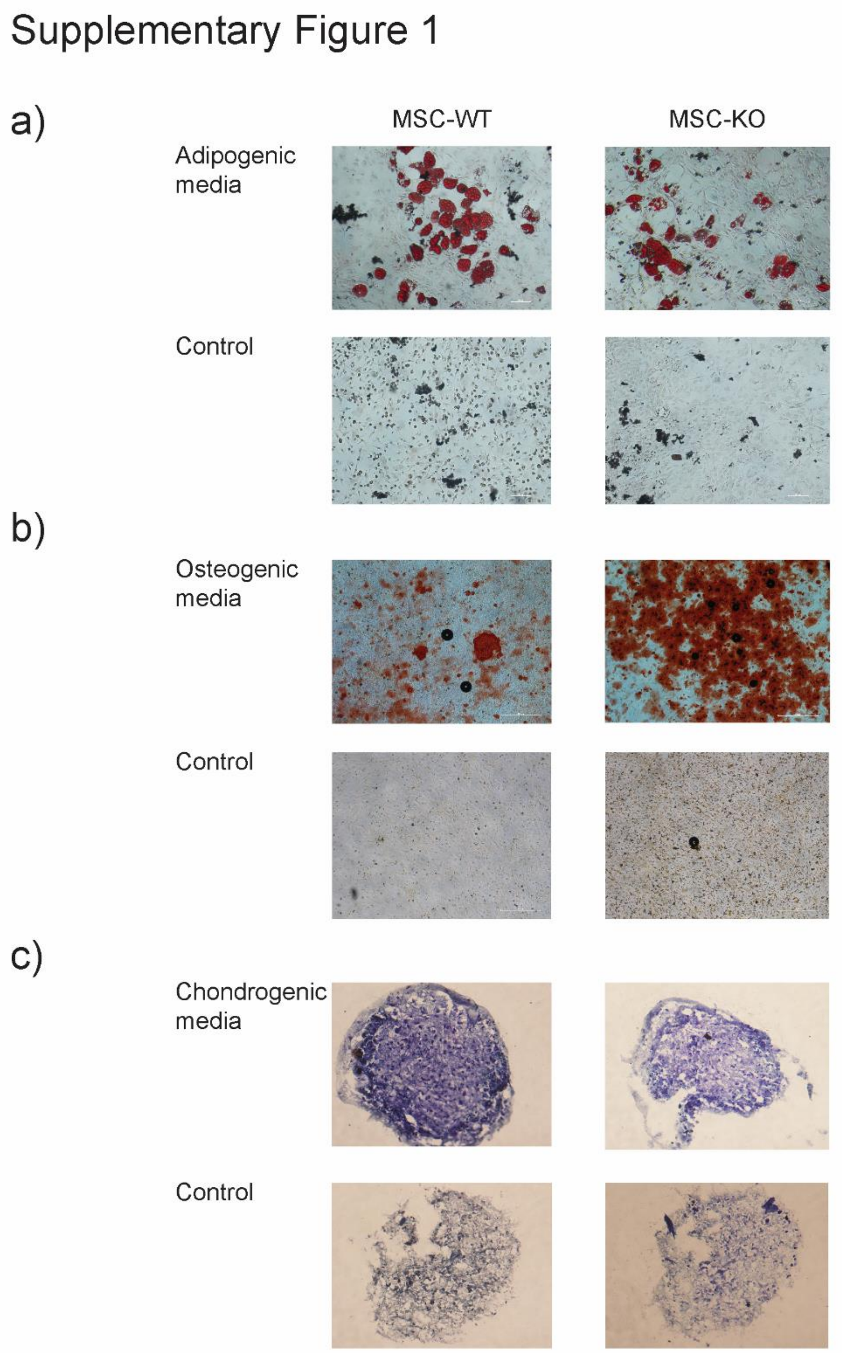

Supplementary Figure 1:

WT MSC and MSC-KO were exposed to various differentiating media and subsequently differentiated into ***A)*** *adipocytes (verified with Oil Red O)* ***B)*** *osteocytes (Alizarin Red stain) and* ***C)*** *chondrocytes (Toluidine Blue stain). Both cell populations were readily able to differentiate into these lineages, while cells cultured in control media did not differentiate.*

Supplementary Figure 2

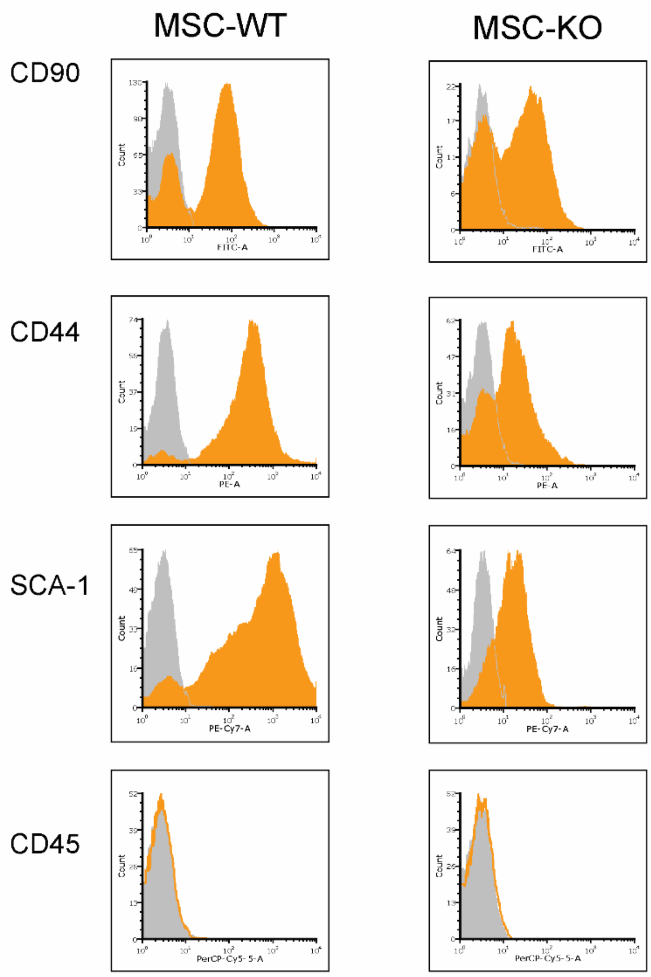

Supplementary Figure 2:

Isolated MSC were characterised for expression of cell surface markers using FACS. Both MSC-WT and MSC-KO were found to express common MSC cell surface antigens CD90, SCA-1 and CD29 (as indicated by orange histograms). MSC were negative for CD45. Control isotype represented in grey histograms.

Supplementary Figure 3

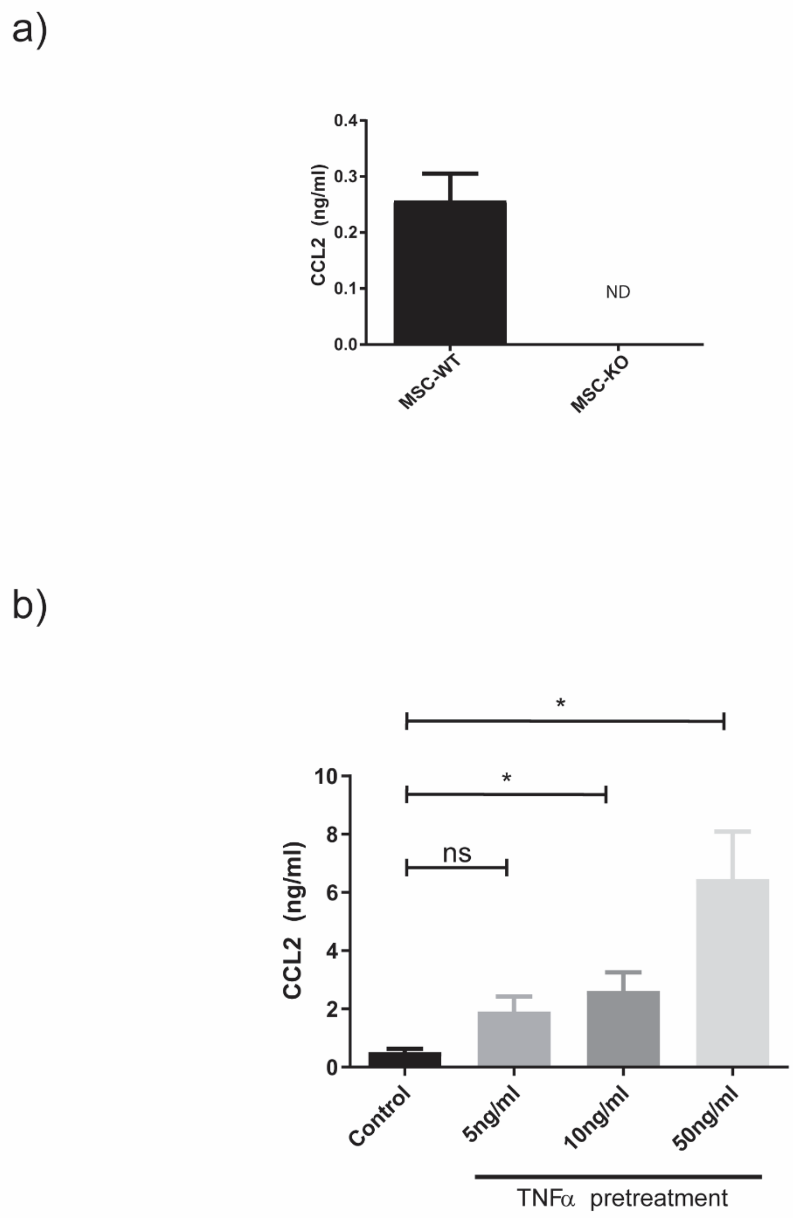

Supplementary Figure 3:

A) *Cells culture supernatants collected at 24h from both MSC-WT and MSC-KO were assayed for CCL2 secretion using ELISA. Under unstimulated conditions MSC secrete CCL2 (0.25ng/ml), no CCL2 was detected in MSC-KO supernatants (n=3).* ***B)*** *Cells culture supernatants collected from MSC-WT stimulated with increasing concentration of TNFα for 24h. Supernatants were assayed for CCL2 using ELISA. TNFα stimulation of MSC-WT increased CCL2 secretion in a dose dependent manner. (n=3, ns, not significant * p<0.05).*

Supplementary Figure 4

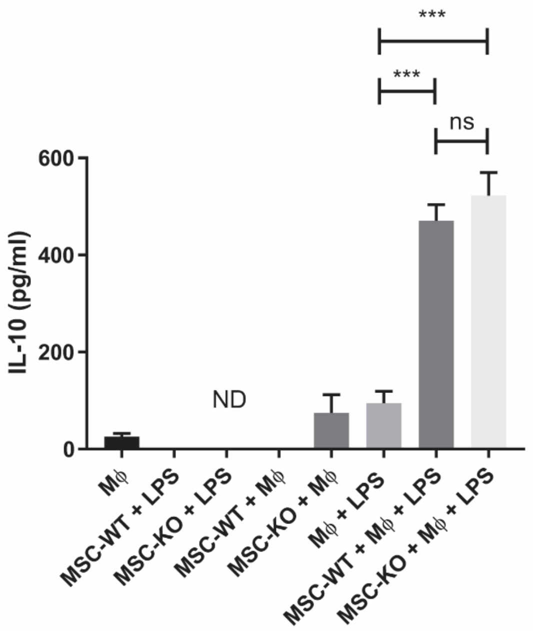

Supplementary Figure 4:

A) *Detection of IL-10 in supernatants demonstrate both MSC-WT and MSC-KO were capable of significantly increasing IL-10, (n=6, ND - not detected, ns - not significant, *** p<0.001)*

Supplementary Figure 5

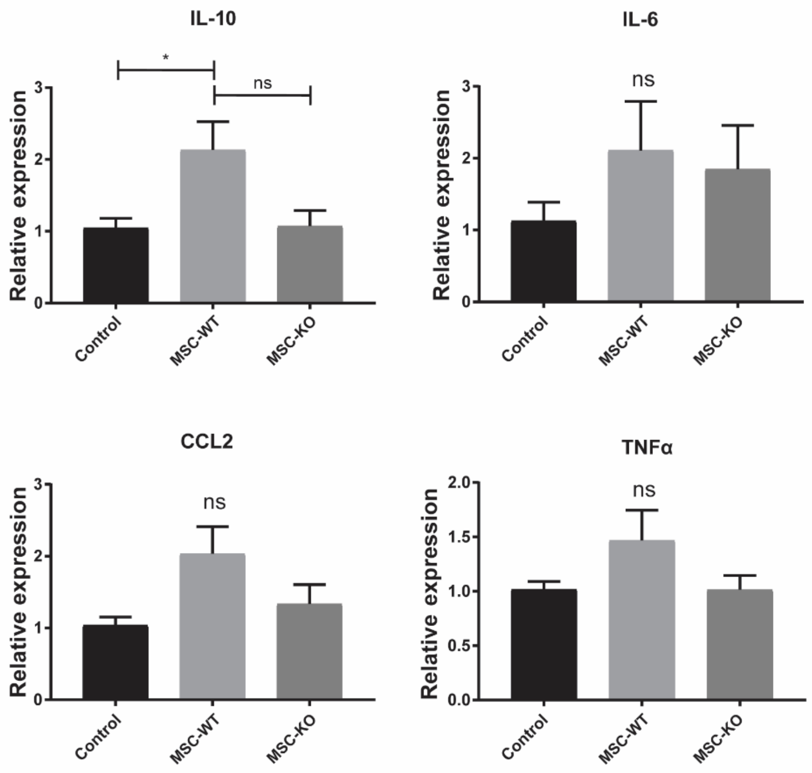

Supplementary Figure 5:

A) *Analysis of cytokine expression (qRT-PCR) from samples containing both wound and healed wound margin at d-10. Application of MSC significantly increased IL-10 associated with macrophage M2 transition, which was abrogated with MSC-KO (n=5, ns, not significant * p<0.05). No difference in either IL-6, CCL2 or TNFα was detected. (n=5, ns, not significant * p<0.05).*

SUPPLEMENTARY METHODS

Flow Cytometry Analysis of Cell Surface Antigens

Expression of associated MSC markers CD44, CD90 and Sca-1 along with CD45 were evaluated to characterise cell population. Cultured cells at P5 were washed once with PBS and detached from the tissue culture flask by trypsinisation followed by neutralisation with equal volume of serum supplemented medium. Cells were then recovered by centrifugation at 500 g for 5 minutes, followed by re-suspension in PBS aliquoted out into several tubes contain $1x10^5$ cells for primary antibody staining. Cells were centrifuged and re-suspended in 100 µl of diluted antibody (see Supplementary Table 1) in FACS buffer and incubated for 1 h at 4°C. Cells were then washed in PBS centrifuged and re-suspended in 100 µl of secondary antibody solution for 30 min in the dark at room temperature (rt). Cells were then re-suspended in 100 µl FACS buffer and analysed on flow cytometer. Control isotypes were used to determine positive cell populations.

Lineage Differentiation assays

MSC phenotype was validated by subjecting BM-MSC isolated from both WT and CCL2 KO mice to in vitro differentiation assays to confirm their potential to differentiate into adipocytes, chondrocytes and osteocytes. Tri-differentiation potential was verified using well established previously published protocols [39, 40].

Briefly, for adipogenic differentiation, cells in culture were subjected to 3 cycles of adipogenic induction media for 3 d followed by adipogenic maintenance media for 1 d. Adipogenic induction media contained dexamethasone 1 mM, insulin 1 mg/ml (Sigma Aldrich, Ireland), indomethacin 100 mM (Sigma Aldrich) and 500 mM MIX (Sigma Aldrich), the adipogenic maintenance media contained insulin 1 mg/ml. Adipogenic differentiation was confirmed with oil red O (Sigma Aldrich, Ireland) staining.

For osteogenic differentiation, cells were cultured in osteogenic differentiation media containing β glycerol phosphate (Sigma Aldrich) 1 M and ascorbic acid (Sigma Aldrich) 2-P 10 mM for 21 d. Osteogenic differentiation was confirmed by Alizarin Red staining.

For chondrogenic differentiation, pelleted cells were cultured for 21 d in chondrogenic differentiation media containing 10 ng/ml TGF-β3 (Sigma Aldrich). Chondrogenic differentiation was confirmed by mounting the pellets in OCT, sectioning them at 10 µm, and staining with toluidine blue (Sigma Aldrich).

ELISA

To determine concentrations of IL-10, TNFα and CCL2, ELISAs were performed on culture supernatants using kits provided by R&D systems (MN, USA). Following manufacturer protocols 96 well clear plastic plates were coated overnight at room temperature with capture antibody (0.2 µg/ml) (R&D systems). 100 µl of diluted culture supernatants was added to the plate for 2 h at room temperature. The plate was washed and detection antibody (50 ng/ml) (R&D systems) applied for 2 h. After washes, streptavidin conjugated horseradish peroxidase (R&D systems) was incubated for 20 min on the plate followed by substrate colour solution (hydrogen peroxide and tetramethylbenzidine) (R&D systems). The reaction was then stopped after 20 min by addition of 100 µl of 2N sulphuric acid (Sigma Aldrich). The optical density was measured using microplate reader (Molecular Devices, Spectra Max, UK) and sample concentration was calculated from a generated standard curve (Soft Max pro v5.4.3, UK). Each standards and sample was measured in duplicate and experiments had multiple replicates.

Re-epithelialisation

Wounds were excised, and trimmed to 1 cm^2. This was bisected and one half was stored in 4% PFA o/n. To determine re-epithelialisation, wound sections were stained with H&E. The wound section was imaged at 4x magnification. Unepithelialised length (or wound gap) was measured using ImageJ software by blinded observers

Granulation tissue area

The area of granulation tissue was assessed from previously stained H&E sections. The area was calculated using image J software to trace the region of granulation tissue from images taken at 4x magnification. An image of a microscope ruler slide taken at the same magnification was used to convert pixels to distance in image J and subsequently granulation area was calculated.

Immunofluorescent staining

Wounds were excised, and trimmed to 1 cm^2 containing both wound and healed wound margins. This was bisected and one half was stored in 4% PFA for 1 h at 4°C, then dehydrated in graded sucrose solution and then embed in OCT. Frozen sections were cut at 10 µm thickness in cryostat and stored at -80°C until required. Prior to staining, slides were removed and left to defrost for 30 min at rt.

Slides were rehydrated in PBS (10 min) and then blocked for 1 h in 10% normal goat serum in PBS-Tween (0.5%) solution. Sections were then incubated o/n at 4°C with primary antibody diluted in antibody buffer (1% BSA PBST). The next day, sections were washed x3 in PBST and slides were incubated with secondary antibody solution in antibody buffer for 2 h at rt, followed by 3x washes in PBST. Nuclei were stained with DAPI (1 µg/ml) for 10 min followed by 3x washes in PBS prior to mounting in immunomount and cover slipping.

Vessel Density quantification

Vessel density was calculated by measuring the quantity CD31 positive vessel like structures within the wound bed from images taken at 40x. Random images were taken within the wound bed through 5 non-consecutive tissue sections. The average quantity of vessels per high power field for each wound (vessel density = vessels per HPF) was then calculated. Average vessel density for each wound was then averaged among the replicate animals.

Supplementary Table 1 Antibodies used in flow cytometry characterisation of murine MSC

Conjugated antibody	Clone	Manufacturer	Final Concentration
CD90-APC-Cy7	53-2.1	BD Pharmingen	2 µg/ml
CD44-PE	IM7	eBioscience	2 µg/ml
SCA-1-PE-Cy7	D7	BD Pharmingen	2 µg/ml
CD45-PerCP-Cy5.5	104	BD Pharmingen	2 µg/ml
Isotype Control-APC-Cy7	R35-95	BD Pharmingen	Matched to primary ab
Isotype Control-PE	eB149/10H5	eBioscience	Matched to primary ab
Isotype Control-PE-Cy7	R35-95	BD Pharmingen	Matched to primary ab
Isotype Control-PerCP-Cy5.5	G155-178	BD Pharmingen	Matched to primary ab

Supplementary Table 2 Immunohistochemistry antibodies used in murine wound sections

Primary antibody	Clone	Manufacture	Final Concentration	Isotype
Ly6G	RB6-8C5	Abcam, UK	5 µg/ml	Rat IgG_{2b}
CD3	SP7	Abcam, UK	1:100	Rabbit IgG
CD31	NA	Abcam, UK	1:100	Rabbit IgG
CD206	NA	Abcam, UK	10 µg/ml	Rabbit IgG
F4/80	CI:A3-1	Abcam, UK	10 µg/ml	Rat IgG_{2b}
Isotype control Rabbit polyclonal IgG	NA	Abcam, UK	Adjusted to primary ab concentration	Rabbit
Isotype control Rat IgG_{2b}	NA	Santa Cruz	Adjusted to primary ab concentration	Rat
**Secondary antibody**	**Clonality**	**Manufacturer**	**Concentration**	**Host**
488 αRabbit IgG (H+L)	polyclonal	Thermo Fisher Scientific	2 µg/ml	Goat
546 αRat IgG (H+L)	polyclonal	Thermo Fisher Scientific	2 µg/ml	Goat
